# Breast Sarcomas: Experience of a Reference Center in Colombia

**DOI:** 10.7759/cureus.5078

**Published:** 2019-07-04

**Authors:** Juan C Vergel, Ana M Osorio, Mauricio Garcia Mora, Óscar García Angulo, Luis Guzmán Abisaab, Sergio Cervera-Bonilla, Sandra Diaz Casas

**Affiliations:** 1 Breast and Soft Tissue Clinic, Instituto Nacional de Cancerologia, Bogotá D.C., COL

**Keywords:** sarcoma, breast neoplasm, surgery, chemotherapy, radiotherapy

## Abstract

Introduction

Breast sarcomas are tumors of a mesenchymal origin, with an incidence of less than 1% of the total breast tumors. The diagnosis of this disease is a challenge for pathologists, radiologists, and breast surgeons.

Aim

To describe the diagnostic, therapeutic, and outcomes approach of patients with breast sarcoma treated at the National Cancer Institute (NCI) in Bogota, Colombia.

Materials and methods

It is a descriptive and retrospective case series study of patients diagnosed with breast sarcoma treated at the NCI during the period between August 1, 2016 and March 30, 2019.

Results

We identified 14 patients diagnosed with breast sarcoma, 10 (71.4%) patients with primary breast sarcomas, and four (28.6%) with sarcomas associated with radiotherapy. The most frequent histological subtype in both, primary and secondary sarcomas, was angiosarcoma (n = 5, 35.7%). 100% (n = 14) of patients received surgical management as primary treatment. Eight (57.1%) patients presented recurrence (disease-free survival (DFS) follow-up of 5.95 months). A total of five deaths were recorded, representing 35.7% of patients (overall survival (OS) follow-up of 23.5 months).

Conclusion

Breast sarcomas are characterized by aggressive clinical behavior, which is why it is important to make a precise histological diagnosis and thus provide patients with radical surgical procedures that ensure local control of the disease and improve DFS.

## Introduction

Breast sarcomas are tumors of a mesenchymal origin, corresponding to less than 1% of all breast tumors and to less than 5% of all sarcomas [1]. According to Surveillance, Epidemiology and End Results (SEER) data, an annual incidence rate of 4.6 cases per 1,000,000 women is calculated. Sarcomas have two origins: they may be breast primary or appear secondary to various associated clinical conditions such as chronic lymphedema, exposure to chest wall radiotherapy, and in patients with genetic disorders such as neurofibromatosis (mutation in NF1gene), Li-Fraumeni syndrome (mutation in TP53 gene) and familial adenomatous polyposis [2-7]. 

Breast sarcomas originate from the periductal stromal and are named according to the type of associated cells. Different histological subtypes have been described, the most frequent is angiosarcoma, representing 0.002% to 0.05% of breast tumors, and a relationship has been identified with radiotherapy received by patients for the treatment of breast cancer, with an adjusted relative risk of 59 times (IC 95% 22-153) [8]; following in frequency, are the variables of fibrosarcomas, leiomyosarcomas, osteosarcomas, liposarcomas, chondrosarcomas, histiocytomas and Kaposi’s sarcoma. Sarcomas have a higher incidence in the female population (98.5%). The incidence of sarcomas of secondary origin is difficult to establish due to its scarce appearance. The average age of diagnosis is between the fifth and sixth decade of life; angiosarcoma of a primary origin appear at younger ages, with an average diagnosis at age 40, while secondary angiosarcoma appears at age 64, due to breast cancer of epithelial origin occurs in this age group [2,9-10]. 

Risk factors for the development of primary sarcomas are not yet established, unlike secondary sarcomas, which are caused by conditions that cause chronic lymphoedema and by exposure to radiotherapy. 

Secondary sarcoma to chronic lymphoedema, was described by Stewart and Treves, this sarcoma occurs in patients who required axillary lymph node dissection or who received radiotherapy at the axillary lymph node chain [6].

Regarding sarcomas associated with radiotherapy, it must be taken into account that the risk of having this disease is directly proportional to the dose of radiation received, and is higher if it was administered during childhood. This risk persists for 20 to 30 years after treatment, with an average appearance at 10 years. The criteria for the diagnosis of sarcomas associated with radiotherapy were established by Cahan, and are the following: first, having a history of malignant tumor of different histology; second, sarcoma developed in irradiated fields; and third, appearing within a latency period greater than four years [5].

Similarly, exposure to certain environmental and biological factors, such as arsenic, vinyl chloride, herbicides, immunosuppressive agents, Kaposi's sarcoma, human immunodeficiency virus (HIV), and human herpes virus type 8, can promote the development of sarcomas [2,5]. 

Clinically, sarcomas are described as a unilateral mass, of variable size (average of 5 to 6 cm), well defined, not painful, solitary, with rapid progression and expansive growth, and in the case of angiosarcomas an involvement of the skin can be evidenced, which appears with papular skin lesions or macules with ecchymosis [11-12]. Regional node involvement is low (less than 5%), and is only reported in certain histological subtypes (clear cells, epithelioid, synovial, angiosarcoma and rhabdomyosarcoma) where it can reach 20% of occurrence [2,9,12-14]. Sarcomas must be staged clinically based on criteria by the American Joint Committee on Cancer (AJCC)[15].

As for diagnosis, findings, and approach, these are performed in the same way as with other diseases of the breast. In mammography, these tumors are described as masses of an oval, defined or irregular margins, and may be reported as distortions of the architecture, not associated with calcifications; likewise, in breast ultrasound, irregular, hypoechoic masses can be observed, with spiculated or indefinite margins. Magnetic resonance imaging (MRI) can help to assess the extent of the disease, showing a mass with indistinct margins and with heterogeneous enhancement, with low signal intensity in T1, but with high intensity in T2, suggesting the presence of vascular channels with slow flow [16-18].

The gold standard of diagnosis in breast sarcomas is the histopathological study, this must always include immunohistochemical markers (IHC) for cytokeratins and myoepithelial cells [19]. Sarcomas are histologically heterogeneous. The differential diagnoses that should be taken into account are ductal breast cancer, phyllodes tumors, lymphomas, extramammary metastases, fibroadenomas, and sclerosing adenosis [2].

The degree of differentiation is the most important prognostic factor; it is for this reason that the pathology study should evaluate cell differentiation, mitotic counting, the presence or absence of necrosis, cellularity and nuclear pleomorphism [2,9]. 

These tumors have the ability to spread by hematogenous dissemination, and the main sites of localization of metastases occur at the lungs, bone, and liver [2,12].

Given its low incidence, there are information gaps when describing its clinical course. Regarding approach and treatment, these have been based on the results obtained through retrospective studies and clinical experience for the treatment of soft tissue sarcomas of the extremities. Surgery is the mainstay of treatment; the type of procedure will depend on the size of the tumor and its relationship with the breast. Section margins greater than 1 cm should be obtained and in angiosarcoma should be 3 cm, since the status of the resection margins is directly related to the survival of patients [3,10,20-21]. Axillary involvement is less than 5%, so its evaluation should only occur when there is a histological subtype that has a higher risk of lymphatic spread or if axillary lymph node involvement is confirmed by biopsy prior to the primary surgical procedure [12,19]. 

Referring to adjuvant treatment, according to data from a meta-analysis conducted in 1997, it was shown that adjuvant chemotherapy based on Doxorubicin and Ifosfamide may have a benefit in disease-free survival (DFS), without finding a statistically significant difference in overall survival (OS) [22]. An update of this meta-analysis, carried out by Peryaiz et al., which included resectable soft tissue sarcomas, found benefit in relation to local recurrence (OR 0.73, 95% IC 0.56 - 0.94, p = 0.03) and distance recurrence (OR 0.67, 95% IC 0.56 - 0.82, p = 0.0001), without finding any difference in the OS (OR 0.56, 95% IC 0.36 - 0.85, p = 5.01) [23]. The recommendation for adjuvant systemic therapy is to initiate it in high-risk patients, defined as tumors of high histological grade, recurrent, larger than 5 cm or with regional nodal involvement [2,23]. Current evidence shows no benefit from the use of neoadjuvant chemotherapy, some studies determine that it can be administered to reduce tumor size; however, the clinical response obtained is low, of around 20%-40%, thus causing delay of surgical resection [24]. 

About adjuvant radiotherapy, its benefit in DFS is not clear, some studies conclude that locoregional recurrence rate decreases and DFS increases, as was evident in the analysis conducted by Johnstone et al., which included 10 patients with non-metastatic primary breast sarcomas who received treatment with mastectomy and adjuvant radiotherapy [25]. Other studies have obtained similar findings, without finding a statistically significant difference between the patients who underwent surgery with radiotherapy versus those who only received surgical management [26-27]. In contrast, some authors recommend it as a method of consolidating adjuvant treatment and reducing the risk of recurrence, based on recommendations from series of cases that show a 5-year reduction in DFS of 64% [28]. According to these outcomes, adjuvant radiotherapy would be indicated in high-grade tumors larger than 5 cm and, in case of presenting positive section margins, this management has been extrapolated from the treatment of extremity sarcomas [23]. 

The prognosis of patients depends on clinical stage of the disease, on a tumor size greater than 5 cm, the histological grade, the margins (which is the most important factor in determining recurrence) and on the histological subtype (being angiosarcoma the worst prognosis). DFS of patients with breast sarcoma varies between 44% and 66%, while OS varies between 49% and 67% [25,29-30]. 

## Materials and methods

A search on the SAP®electronic medical records system (SAP GUI for Windows Version 7400.1.0.1093, Walldorf, Germany) of patients with a diagnosis of breast sarcoma, was carried out. We found 14 patients who were treated at the National Cancer Institute (NCI) in Bogota, Colombia during the period between August 1, 2016 and March 31, 2019. Survival functions were estimated for each of the two outcomes (OS and DFS) using the Kaplan-Meier method. 

This study was approved by the institutional ethics committee and was classified as a risk-free study. 

## Results

The median age of patients was 51.2 years (19-86 years). Of these patients, 10 (71.4%) had primary breast sarcomas. The most frequent histological subtype, both in primary and secondary sarcomas, was angiosarcoma (n = 5, 35.7%). Regarding the diagnosis of secondary sarcoma associated with radiation for adjuvant treatment of breast cancer, the latency period varied between 5 and 9 years, with a mean occurrence of 6.7 years. The most frequent clinical stage found was IIIA (n = 5, 35.7%); and only one patient (7.1%) presented axillary lymph node involvement. 78.6% (n = 11) of tumors had a high grade of differentiation (Table 1).

**Table 1:** Clinical and histological characteristics of patients with breast sarcomas at the NCI Breast Functional Unit. NA, not applicable

Case	Age (years old)	Histological type	Origin	Clinical stage	Histological grade	Tumor size (cms)	Lymph node involvement	Metastatic involvement
1	27	Stromal	Primary	IB	Low grade	6.5 x 5.5 x 4	NA	No
2	70	Fusocellular and myxoid	Primary	IIIA	High grade	8 x 6 x 4	NA	No
3	56	Osteoblastic and fibroblastic osteosarcoma	Primary	IIIA	High grade	6 x 6 x 2.5	NA	No
4	86	Epithelioid angiosarcoma	Primary	IIIA	High grade	9 x 5 x 7	0/21	No
5	64	Fibroblastic fusocellular - Myofibroblastic	Primary	IV	High grade	22 x 18 x 20	NA	Lungs
6	23	Pleomorphic liposarcoma	Primary	IIIB	High grade	16 x 13 x 10	NA	No
7	36	Epithelioid angiosarcoma	Primary	IB	Low grade	8 x 9 x 3.8	0/9	No
8	34	Dermatofibrosarcoma protuberans	Primary	IA	Low grade	3.5 x 3 x 3.5	NA	No
9	19	Epithelioid angiosarcoma	Primary	IIIB	High grade	14 x 14 x 14	0/1	No
10	51	Fusocellular and pleomorphic	Primary	IV	High grade	22 x 22 x 11	27/34	Liver
11	71	Epithelioid angiosarcoma	Secondary	II	High grade	1.5 x 1.5 x 0.5	NA	No
12	72	Epithelioid angiosarcoma	Secondary	IIIB	High grade	20 x 11 x 11	NA	No
13	62	Pleomorphic spindle cell with an osteochondromatosis component	Secondary	IIIA	High grade	6 x 5 x 5	NA	No
14	47	Carcinosarcoma	Secondary	IIIA	High grade	10 x 7 x 5	NA	No

Referring to the treatment received by patients, 100% (n = 14) of patients received surgical management as a primary treatment, with simple mastectomy the mostly performed procedure (n = 8, 57.1%). For adjuvant therapy, only 14.3% (n = 2) of the patients received chemotherapy, while six patients (42.9%) received radiotherapy. Of the total of 14 patients, five (35.7%) required intervention by the Plastic Surgery service for breast reconstruction or covering the surgical defect (Table 2).

**Table 2:** Treatments administered to patients diagnosed with breast sarcomas at the NCI Breast Functional Unit. Gy: gray

Case	Primary surgery	Adjuvant chemotherapy	Adjuvant radiotherapy	Type of reconstruction or covering
1	Simple mastectomy	No	50 Gy	Reconstruction with expander
2	Simple mastectomy	No	No	No
3	Simple mastectomy	No	50 Gy	No
4	Radical modified mastectomy	No	No	No
5	Hygienic simple mastectomy	Doxorubicin + Ifosfamide x 4 cycles	50 Gy	No
6	Radical modified mastectomy	No	No	Reconstruction with latissimus dorsi muscle + expander / prosthesis
7	Simple mastectomy + sentinel ganglion	No	50 Gy	Reconstruction with latissimus dorsi muscle + definitive prosthesis
8	Local wide breast resection	No	No	No
9	Simple mastectomy	No	60 Gy	No
10	Hygienic radical modified mastectomy	No	No	Covering with latissimus dorsi muscle
11	Simple mastectomy	No	No	No
12	Hygienic simple mastectomy	No	No	No
13	Wide local resection + cleidectomy + partial sternectomy + chest wall reconstruction	Doxorubicin + Cisplatin x 4 cycles.	No	No
14	Local wide breast resection + partial cleidectomy	No	40 Gy	TRAM flap covering

**Figure 1:**
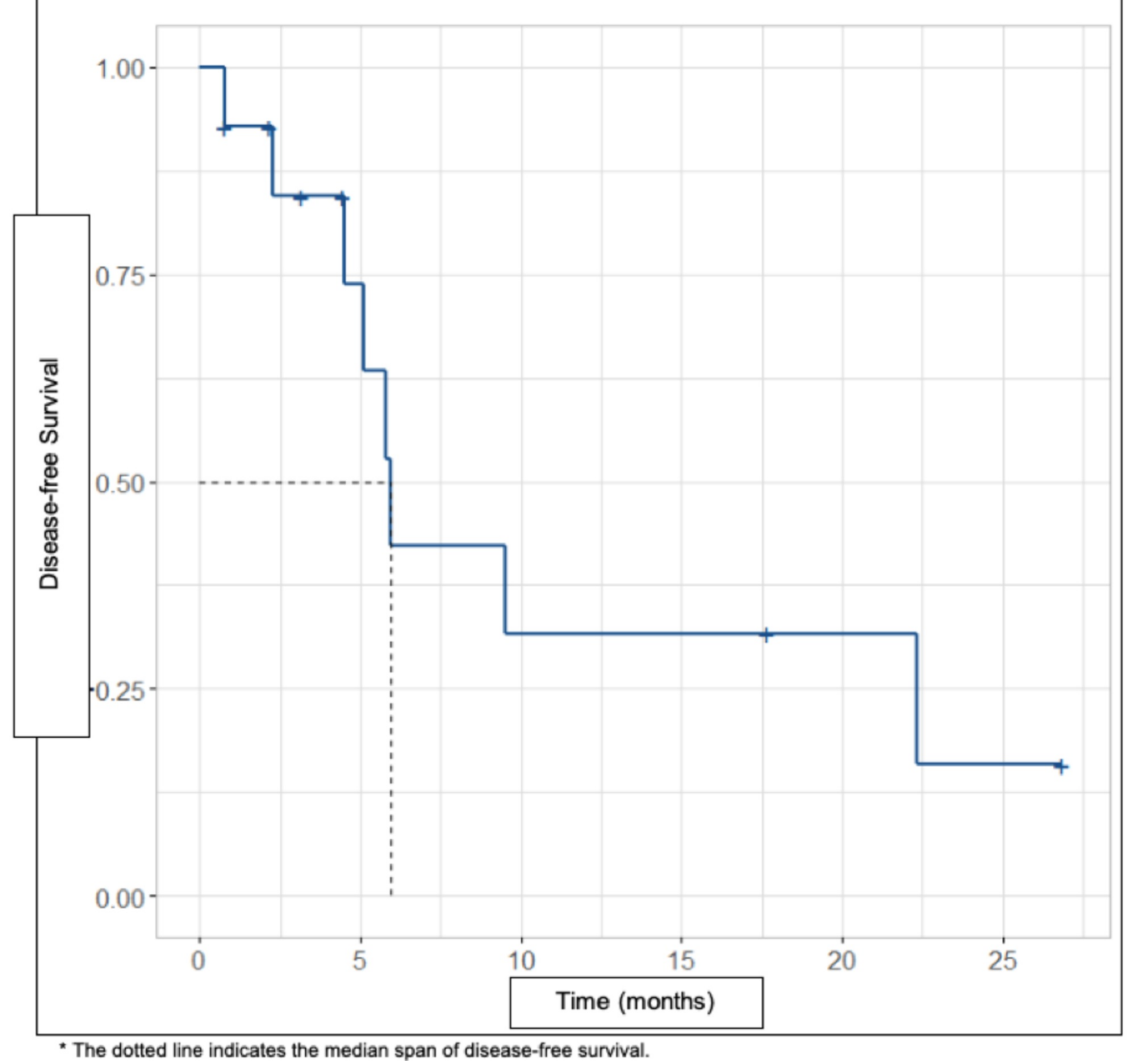
Disease-free survival curve by Kaplan-Meier method (blue line).

**Figure 2:**
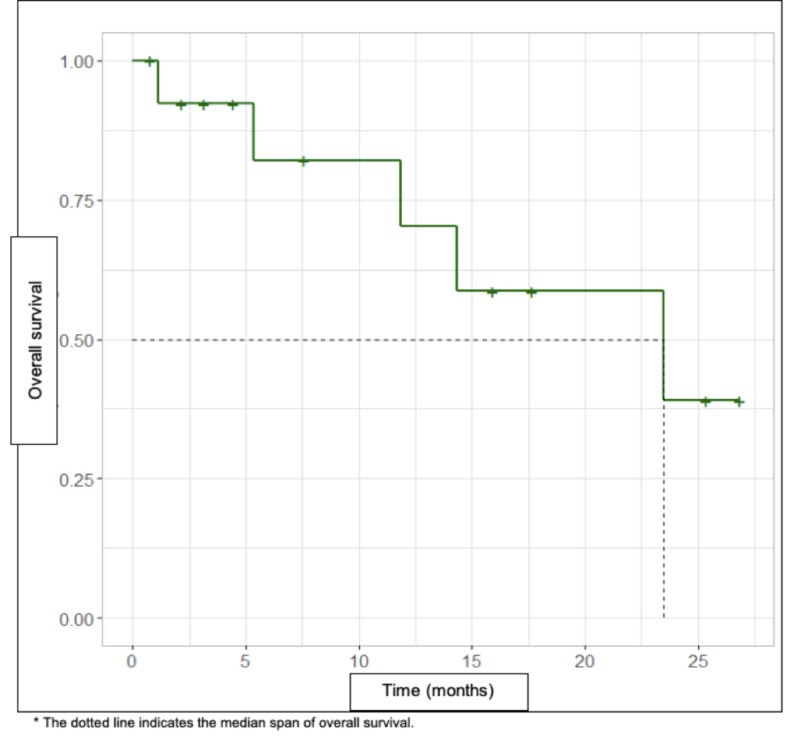
Overall survival curve by Kaplan-Meier method (green line).

Eight (57.1%) patients relapsed, representing 75.0% (n = 6) of patients at a local recurrence (DFS follow-up of 5.95 months) (Figure 1). There were five (35.7%) deaths of patients in this series of cases and the remaining nine are alive without disease (64.3%) (OS follow-up of 23.5 months) (Figure 2). It was found that the deceased patients, in their entirety, had local recurrence (n = 4), regional recurrence (n = 1, soft tissue) and systemic recurrence (n = 4, lungs) (Table 3). 

**Table 3:** Treatment of relapses and vital status of patients with breast sarcomas at the NCI Breast Functional Unit. NA: not applicable

Case	Presented relapse	Location of relapse	Surgery	Chemotherapy	Radiotherapy	Vital Status
1	No	NA	NA	NA	NA	Alive
2	Yes	Local	Wide local resection + resection of the 4^th^ and 5^th^ costal arch	Doxorubicin + Ifosfamide x 4 cycles	50 Gy	Alive
3	No	NA	NA	NA	NA	Alive
4	No	NA	NA	NA	NA	Alive
5	No	NA	NA	NA	NA	Alive
6	Yes	Local - Lungs	Wide local resection	No	No	Dead
7	No	NA	NA	NA	NA	Alive
8	Yes	Local	Wide local resection + Mohs surgery	NA	NA	Alive
9	Yes	Regional (right infraclavicular ganglion)	Wide local resection	Doxorubicin + Ifosfamide x 4 cycles	No	Alive
10	Yes	Local - Lungs	NA	NA	NA	Dead
11	No	NA	NA	NA	NA	Alive
12	Yes	Local	No	Paclitaxel x 2 cycles	No	Dead
13	Yes	Local - Regional (soft tissues, right muscle and paratracheal) - Lungs	No	Ifosfamide x 1 cycle	No	Dead
14	Yes	Lungs	Right inferior lobectomy + right upper lobe wedge + regional nodes resection	Gemcitabine + Dacarbazine x 1 cycle	No	Dead

## Discussion

Due to the low incidence of breast sarcomas, it is very difficult to perform a prospective study on patients with this diagnosis, the vast majority of the studies found in the literature are reports and case series. The biological behavior of breast sarcomas is similar to that of sarcomas originating in the extremities, presenting the same prognostic factors such as histological grade, tumor size, and nodal involvement [2,23]. The majority of recurrences occurred at a local level; and when distant metastases were present, the main organs affected were the lungs [2,12]. 

Comparing with the Bousquet study [9], where distant metastases were reported in 36% of the population, similarity was found with our series of cases, in which the distance involvement was presented in 28.6% (n = 4) of the patients; additionally in both studies the organ with the highest frequency of involvement was the lungs with 70% and 100%, respectively.

According to international literature, these tumors present a high risk of recurrence (DFS: 44%-66% at 5 years) and mortality (OS: 49%-67% at 5 years) [3,9,25,29-30], this is consistent with our study in which we found DFS rates of 42.9% with follow-up at 5.95 months and OS of 64.2% with follow-up at 23.5 months.

The prognosis of this disease is poor, due to its difficult diagnosis, surgical management, adjuvant treatment and high risk of recurrence [25,29-30]. 

## Conclusions

Breast sarcomas are very low incidence tumors so it is important to recognize their clinical and biological behavior. This case series study shows that this disease has a very aggressive behavior; therefore, it is important to make an adequate histological diagnosis for being able to offer priority treatment to patients, having surgical management as a pillar thereof. Regarding adjuvant treatment, current clinical practice guidelines do not recommend offering routine chemotherapy or radiotherapy, and in many cases, these therapeutic options are relegated to the management of tumor recurrences.
